# Mixed Mutual Transfer for Long-Tailed Image Classification

**DOI:** 10.3390/e26100839

**Published:** 2024-10-02

**Authors:** Ning Ren, Xiaosong Li, Yanxia Wu, Yan Fu

**Affiliations:** College of Computer Science and Technology, Harbin Engineering University, Nantong Street, Harbin 150001, China; ning.ren.hrbeu@outlook.com (N.R.); lixiaosong@hrbeu.edu.cn (X.L.); fuyan@hrbeu.edu.cn (Y.F.)

**Keywords:** convolutional neural network, imbalanced learning, long-tailed image classification, rebalancing

## Abstract

Real-world datasets often follow a long-tailed distribution, where a few majority (head) classes contain a large number of samples, while many minority (tail) classes contain significantly fewer samples. This imbalance creates an information disparity between head and tail classes, which can negatively impact the performance of deep networks. Some transfer knowledge techniques attempt to mitigate this gap by generating additional minority samples, either through oversampling the tail classes or transferring knowledge from the head classes to the tail classes. However, these methods often restrict the diversity of the generated minority samples, primarily focusing on transferring information only to the tail classes. This paper introduces a simple yet effective method for long-tailed classification, called mixed mutual transfer (MMT), which facilitates the mutual transfer of knowledge between head and tail classes by blending samples. The core idea of our method is to create new samples by blending head and tail samples. Head samples are selected using a uniform sampler that retains the long-tailed distribution, while tail samples are selected using a differential sampler that reverses the long-tailed distribution to alleviate imbalance. Our approach aims to diversify both tail and head classes. During the training phase, we utilize the generated samples to update the original dataset for training deep networks. Mixed mutual transfer simultaneously enhances the performance of both head and tail classes. Experimental results on various class-imbalanced datasets show that the proposed method significantly outperforms existing methods, demonstrating its effectiveness in improving the performance of long-tailed deep networks.

## 1. Introduction

**Figure 1:**
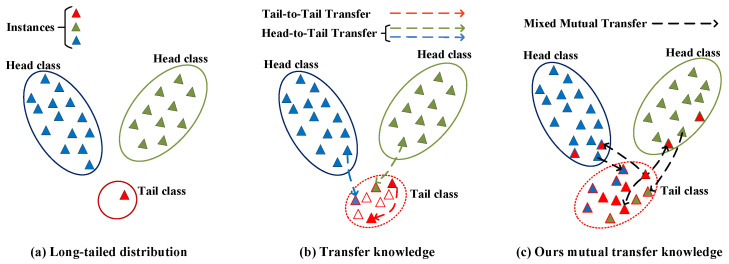
The diagrams of transfer methods. (**a**) Long-tailed distribution, (**b**) oversampling the tail classes or transferring the information from head to tail, and (**c**) mutually transferring information between head and tail. Our key idea is to mutually transfer the knowledge between head and tail samples. More details are presented in Section 3.

Deep neural networks demonstrate exceptional performance on balanced datasets across various visual tasks, such as image classification and object detection. However, real-world datasets often exhibit imbalanced and long-tailed class distributions [1,2,3]. In these distributions, the majority categories (Head) occupy most of the data, while minority categories (Tail) have very few samples [4,5], as shown in Figure 1a. For instance, in an animal recognition task, collecting data on butterflies is relatively easy, whereas gathering data on lizards is much more challenging. Rare and dangerous animals cannot be collected in quantities comparable to more common species [6]. Long-tailed datasets present significant challenges for training deep neural networks [7,8,9], particularly when the objective is to achieve balanced performance metrics in practical applications [1,10].

Numerous methods have been introduced for long-tailed classification tasks [11,12,13,14]. A straightforward approach to address the imbalanced problem is tail-to-tail transfer learning, as shown in Figure 1b. For example, SMOTE [15] oversamples the tail samples by generating additional tail samples, which are created by synthetic samples along line segments connecting existing tail samples. Then, several variants of SMOTE are suggested accordingly [16,17,18], where ADASYN [16] utilizes a weighted distribution of tail class instances to generate additional tail samples. Although these oversampling methods transfer knowledge from tail classes, they often lead to overfitting due to the limited representation of tail samples. Instead of transferring from the tail classes, some methods transfer knowledge from the head to the tail classes to generate more tail samples, namely head-to-tail transfer as illustrated in Figure 1b. For instance, M2m [13] translates head samples into tail samples using another classifier independently trained under the imbalanced dataset. The approach H2T-FAST [19] randomly fuses the style information of the head samples and the content of the tail samples to generate new tail data. These methods enable the long-tailed deep networks to learn a better solution than the tail-to-tail transfer. However, these methods are exclusively oriented towards the generation of tail samples, thereby neglecting the head classes. It is imperative to acknowledge that both head and tail classes play an integral role in model training, which represents the core objective of our optimization.

This paper introduces a robust knowledge transfer method, mixed mutual transfer (MMT), which maximizes the mutual information between head and tail classes by directly mixing samples, thereby simultaneously enhancing the performance of all classes as shown in Figure 1c. Typically, mixed samples are generated by combining random images with their corresponding label pairs, enabling the network to learn from a broad set of virtual training samples and enhancing the model’s generalization ability [20]. However, in long-tailed tasks, this approach of directly mixing random images with their labels can worsen the imbalance [21,22]. To further explore the potential of sample blending in long-tailed tasks, we propose an innovative bi-directional knowledge transfer mechanism that facilitates knowledge sharing between head and tail samples. Specifically, we employ a specially designed sample blending strategy in which new samples are generated by linearly interpolating two samples selected from a uniform sampler and a differential sampler. The uniform sampler preserves the long-tailed distribution of the original dataset by sampling each instance with equal probability, while the differential sampler reverses the distribution of head and tail classes by assigning sampling probabilities based on the difference between the maximum class size and the size of each class. In the mixing process, the two samples are distorted to increase the diversity of the knowledge transferred, resulting in new training samples that increase the variability of both the tail and head classes. During neural network training, the proposed method updates the dataset online, exploring a more diverse data space to enhance the generalization ability in long-tailed learning. Notably, the knowledge transfer occurs not only from the head classes but also from the tail classes, encompassing the entire dataset, which effectively improves the performance across all classes.

In summary, our contributions are threefold:

- We propose a novel transfer augmentation method, mixed mutual transfer, which enhances the performance of both minority and majority classes by maximizing the mutual information between head and tail classes.
- We propose a differential sampler that reverses the long-tailed distribution of the original dataset, reducing extreme imbalance and enhancing the classification of tail classes.
- Experimental results on the CIFAR100-LT, CIFAR10-LT, Tiny ImageNet-LT, and Food101-LT benchmarks demonstrate that our method outperforms the baseline (CE) by 6.12%, 8.73%, 4.27%, and 6.73%, respectively, with an imbalance ratio of 100.

## 2. Related Work

In this section, we review the key approaches to long-tail learning, focusing on transfer knowledge methods (including tail-to-tail transfer learning and head-to-tail transfer learning), generalization learning, and re-weighting methods.

### 2.1. Tail-to-Tail Transfer Learning

Oversampling is one of the most common deep learning methods [23,24]. A straightforward way to balance the training batch is the random oversampling strategy, which emphasizes the tail classes and increases the instance number of the tail classes [25]. For example, SMOTE [15] generates tail-class samples by mixing several intraclass neighboring samples. Following the success of SMOTE, several variants have been developed: Borderline-SMOTE [17], which oversamples the minority samples near class borders, and Safe-level-SMOTE [26], which defines safe regions not to oversample samples from different classes. These methods have been widely used by classical machine learning algorithms because since the tail samples are repeatedly drawn, it can lead to overfitting. Generative Adversarial Minority Oversampling (GAMO) [18] addresses this concern by generating fresh minority samples through the training of a convex generator, a concept inspired by the achievements of Generative Adversarial Networks (GANs) [27] in image generation. Nevertheless, the generator training entails a significant additional training cost, and GAMO may be susceptible to the well-known issue of mode collapse observed in GANs [28].

### 2.2. Head-to-Tail Transfer Learning

Some studies [29,30,31] address the data scarcity of minority categories by head-to-tail transfer learning. Head-to-tail transfer learning seeks to transfer the knowledge from head categories to augment tail classes. For example, M2m [13] transfers knowledge from majority classes using a pre-trained network, and Balancing Long-Tailed datasets (BLT) [32] uses a gradient-ascent image generator to obtain adversarial image generation to compensate for the imbalance in a long-tailed dataset. In recent research, CMO [22] chooses CutMix [33] to generate mixed samples by pasting minority-class images onto rich-context majority-class images, using the latter as backgrounds. Bi-F3R [34] transfers the representation space of head-class to tail-class information to improve the tail-class performance. Nevertheless, these transfer methods [35,36] bolster the performance of tail classes, but they do so at the cost of the intricate model and module designs for knowledge transfer, which may complicate the model’s training and convergence [8]. Unlike these methods, our approach realizes knowledge interaction without introducing complex models and enhances the performance of the tail class without compromising that of the head class.

### 2.3. Generalization Learning

Some studies [29,30,31,37] address the data scarcity of minority categories by introducing augmentation methods. Mixup [20] augmentation improves the generalization of state-of-the-art neural network architectures. This scheme has been applied to solve the problem of long-tailed identification [21,29,35,38]. Remix [21] endeavors to adjust the blending factor of Mixup to suit long-tailed tasks. MixSKD [39] integrates self-knowledge distillation with Mixup, performing mutual distillation between original and Mixup images by aligning feature maps and probability distributions, thereby improving robustness and generalization. MCL [40] enhances feature representations by transferring contrastive distributions across networks through an adaptive, meta-learning-optimized layer-matching mechanism, enabling networks to acquire additional knowledge and improve visual recognition performance. In contrast, MMT focuses on long-tailed image classification and explores diverse mixed samples, building on the Mixup framework. MMT promotes knowledge interaction between majority and minority classes on a global scale, generating more diverse and representative samples, ultimately enhancing the classifier’s generalization ability.

### 2.4. Re-Weighting Methods

Re-weighting aims to provide different weights for samples of different categories and set a more significant weight for samples of the tail categories [11,41]. On the one hand, based on the sample influence or model prediction alignment with the balanced reference distribution [42], further research methods improve loss by adjusting the impact of tag frequency on the loss weight [43]. Another line of the study is class-balanced loss (CB) [41], which introduced a novel concept of effective numbers to approximate the expected sample number of different categories. This scheme measures whether data overlap by associating each sample with a small neighborhood rather than a separate sample point. Following this concept, the CB loss incorporates a category balance re-weighting term, which is inversely proportional to the number of effective categories, to address the issue of category imbalance. Focal loss [44] uses the prediction probabilities to inversely re-weight categories to assign higher weights to the more challenging tail categories but lower weights to the more accessible head categories. Meta-weight-net [45] automatically learns an explicit loss-weight function parameterized by an MLP from data in a meta-learning manner. Due to the universal approximation capability of this weight net, it can finely fit a wide range of weighting functions, including those used in conventional research. In short, typical re-weighting methods often assign a greater weight to the loss of tail predictions, potentially overlooking valuable loss information from head samples. In contrast, our proposed method updates the original dataset with newly generated samples, allowing the model to fully leverage knowledge from both head and tail samples without losing information.

## 3. Proposed Method

In this section, we introduce our novel approach called mixed mutual transfer (MMT). We lay the theoretical foundation for MMT, inspired by the concept of vicinal risk minimization. We also describe the design of our data samplers. In particular, we detail how the uniform and differential samplers work together to address class imbalance in long-tailed datasets. This collaboration facilitates effective knowledge transfer between predominant (head) and rare (tail) classes, thereby improving overall classification performance.

### 3.1. From Vicinal Risk Minimization to MMT

The principle of vicinal risk minimization (VRM) [46] involves generating virtual samples from a distribution around the training data, approximating the actual data distribution. Specifically, let *x* denote a training sample, and *y* is its corresponding label. The objective risk under VRM can be expressed as follows:

(1) $$\begin{array}{c} R_{\nu }\left(f\right)=\frac{1}{m}\sum_{i=1}^{m}\ell \left(f\left(x_{i}\right),y_{i}\right) \end{array}\;$$

where $\left(x_{i},y_{i}\right)$ are virtual samples drawn from a vicinal distribution. One implementation of VRM is the mixup [20], which creates new virtual samples using the following formula:

(2) $$\begin{array}{cc} \; & \tilde{x}=\lambda x_{i}+\left(1-\lambda \right)x_{j},\\ \; & \tilde{y}=\lambda y_{i}+\left(1-\lambda \right)y_{j}, \end{array}\;$$

where $\left(x_{i},y_{i}\right)$ and $\left(x_{j},y_{j}\right)$ are randomly chosen from the training dataset, it does not take into account the majority or minority class from long-tailed distribution. To adapt it to the long-tailed dataset, we absorb the concept of majority and minority samples into Mixup.

**Figure 2:**
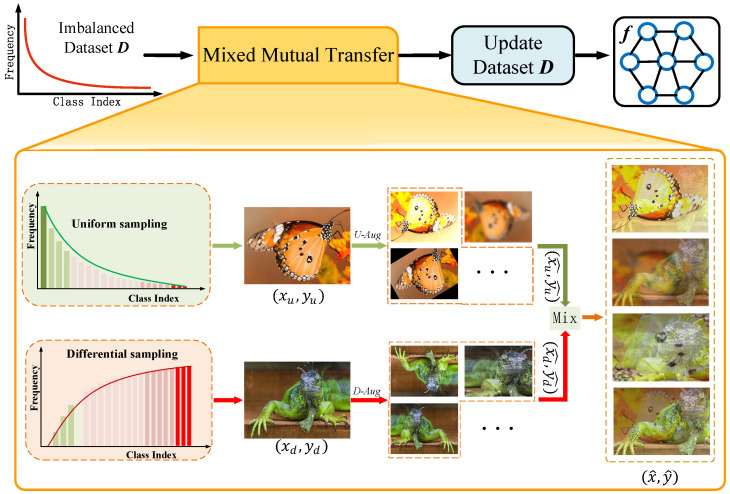
The framework of our mixed mutual transfer (MMT).

The contribution of this paper is to propose a balanced method called mixed mutual transfer (MMT) to augment imbalanced datasets and improve the performance of deep networks on long-tailed distributions. MMT adopts blending samples to mutually transfer knowledge between head and tail classes without relying on a complex transfer model, like M2m [13]. The blending samples are obtained by linearly interpolating with the head images and tail images. As shown in Figure 2, let $x\in R^{W\times H\times C}$ and *y* denote a training sample and its label, respectively. We aim to generate a new sample $\left(\hat{x},\hat{y}\right)$ by linearly interpolating two samples $\left(x^{u},y^{u}\right)$ and $\left(x^{d},y^{d}\right)$. Here, the head image $x^{u}$ carries the knowledge of the majority classes, and the tail image $x^{d}$ carries the knowledge of the majority classes. After combination, the new sample contains knowledge mutually transferred from between head and tail classes.

For the mixed image method, we resort to the augmentation method Mixup [20] due to its simplicity and effectiveness. Moreover, we augment the head images and tail images to increase the diversification of blending samples because we adopt new blended samples for supplementing the original dataset. Here, we adopt different augmentation strategies for head and tail images. Since the sample size of the head class occupies the most, we augment the head images by a simple augmentation method to avoid the augmentation semantic ambiguity and augment the tail class using a strong augmentation method to increase the diversification of information. Following Mixup settings with sample augmentation, the head and tail image and label are blended as

(3) $$\begin{array}{cc} \; & \lambda ∼\mathrm{Beta}(\alpha ,\alpha ),\\ \; & \hat{x}=\lambda \cdot \mathrm{DAug}\left(x_{d}\right)+\left(1-\lambda \right)\cdot \mathrm{UAug}\left(x_{u}\right),\\ \; & \hat{y}=\lambda \cdot \mathrm{DAug}\left(y_{d}\right)+\left(1-\lambda \right)\cdot \mathrm{UAug}\left(y_{u}\right), \end{array}\;$$

where the DAug and UAug represent strong augmentation and simple augmentation, respectively. Here, we adopt RandAugment [47] for DAug and TrivialAugment [48] for UAug in practice. The mixing ratio λ∼Beta(α,α) controls the interpolation strength between sample pairs, where α∈(0,∞). An experiment on using a different α is included in the ablation Section 4.4.3.

Since Mixup is primarily designed for datasets with a uniform class distribution [49,50], the two samples for blending are equally picked up from the dataset, which degrades the performance of deep networks in long-tailed datasets. Therefore, we sample the head images from a uniform sampler, which retains the long-tailed distribution of the original dataset, and the tail images from a differential sampler to be biased to the tail classes. The pseudo-code for training deep networks is presented in Algorithm 1.
**Algorithm 1** Training process of MMT**Input:** A class-imbalanced dataset *D*, uniform sampler $P^{u}$, differential sampler $P^{d}$.**Output:** A model *f*. 1:Initialize the model *f*; 2:**for** each epoch **do** 3:    // Update the dataset. 4:    $D_{g}=\left\{\right\}$; 5:    **for** k=1 to *K* **do** 6:        $\Delta \leftarrow N_{1}-N_{k}$                  ▹$N_{1}$ is the largest category; 7:        **for** i=1toΔ **do** 8:           // Sampling 9:           $(x_{d},y_{d})\leftarrow$ Sample from the differential sampler $P^{d}$; 10:         $(x_{u},y_{u})\leftarrow$ Sample from the uniform sampler $P^{u}$; 11:           λ←Beta(α,α); 12:           $\hat{x}=\lambda \cdot \mathrm{DAug}\left(x_{d}\right)+\left(1-\lambda \right)\cdot \mathrm{UAug}\left(x_{u}\right)$; 13:           $\hat{y}=\lambda \cdot \mathrm{DAug}\left(y_{d}\right)+\left(1-\lambda \right)\cdot \mathrm{UAug}\left(y_{u}\right)$; 14:           $D_{g}\leftarrow D_{g}\cup \left(\hat{x},\hat{y}\right)$; 15:        **end for** 16:    **end for** 17:    $D_{T}\leftarrow D\cup D_{g}$; 18:    Training *f* using dataset $D_{T}$; 19:**end for**

### 3.2. Data Sampler

To sample the head classes and tail classes, we designed the uniform sampler and differential sampler. The uniform sampler retains the long-tailed distribution of the original dataset, where each sample in the training dataset is sampled with equal probability. The differential sampler reverses the head–tail distribution, where the sampling possibility of each class is proportional to the differential between the maximum class size and its sample size.

Considering a long-tailed training dataset $D\in \{D_{1},D_{2},$$\cdots ,D_{K}\}$, where $D_{K}$ is the sub-dataset that exclusively contains samples labeled with *k*, and K denotes the number of categories. The number of the sub-dataset is represented as $N\in \{N_{1},N_{2},\cdots ,N_{K}\}$. Without loss of generality, it is assumed that $N_{1}\geq N_{2}\geq \ldots \geq N_{K}$. As shown in Figure 2, the sample $\left(x_{u},y_{u}\right)$ is taken from a uniform sampler with replacement. The uniform sampler retains the long-tailed distribution of the original dataset, where the sampling probability $P^{u}$ for class k is determined as follows:

(4) $$P_{k}^{u}=\frac{N_{k}}{\sum_{i=1}^{K}N_{k}}.$$

Meanwhile, the sample $\left(x_{d},y_{d}\right)$ is picked up from a differential sampler with replacement. The differential sampler reverses the long-tailed distribution to alleviate the extreme imbalance and improve the classification of tail classes, where the sampling probability $P^{d}$ for class k is determined as follows:

(5) $$P_{k}^{d}=\frac{N_{1}-N_{k}}{\sum_{i=1}^{K}\left(N_{1}-N_{k}\right)},$$

where $N_{1}$ refers to the sample size of sub-dataset $D_{1}$, which has the maximum sample size in all sub-datasets.

## 4. Experiments

In this section, we evaluate the effectiveness of our proposed method through a series of empirical studies, including the Experimental Setup, Main Results, Further Analysis, and Ablation studies.

### 4.1. Experimental Setup

Datasets. We verify the effectiveness of our method on four commonly used benchmark datasets: CIFAR-10, CIFAR-100 [38], Tiny ImageNet [51], and Food101 [52]. The CIFAR-10 and CIFAR-100 datasets consist of 50,000 training images and 10,000 test images with 10 and 100 categories, respectively. Tiny ImageNet is an image classification dataset provided by Stanford University. It contains 200 categories, each containing 500 training images, 50 validation images, and 50 test images. Food101 is a food image classification dataset that is challenging for real-world applications. The Food101 dataset consists of 101 food categories with 750 training and 250 test images per category, making a total of 101 k images. Notably, the image sizes within the Food101-LT dataset are randomized, posing a greater challenge. Moreover, the test image labels have undergone manual cleaning, whereas the training set may contain some noise.

Following [53], we modify the balanced CIFAR-10, CIFAR-100, TinyImageNet, and Food101 datasets to the uneven setting (named CIFAR-10-LT, CIFAR-100-LT, TinyImageNet-LT, and Food101-LT) by utilizing the exponential decay function $n_{k}\mu^{k}\left(\mu \in \left(0,1\right)\right)$, where $n_{k}$ is the original number of the k−th class. The degree of category imbalance in these datasets is represented by the imbalance ratio $IF=\frac{\max_{i}N_{i}}{\min_{j}N_{j}}$, where $N_{k}$ is the number of training samples in the k−th class. We conduct experiments on CIFAR-10-LT, CIFAR-100-LT, TinyImageNet-LT, and Food101-LT using different imbalance ratios: {100, 50, 10} (Table 1).

Complexity analysis. MMT mutually transfers knowledge between head and tail classes by directly blending samples. Compared to multi-stage training methods such as ResLT, DiVE, and LFME, our approach is both time-saving and more simple. This is attributed to its achievement of mutual knowledge transfer between head and tail classes through direct sample blending using uniform and differential samplers, eliminating the need for auxiliary models or multiple classification heads. While re-weighting methods avoid additional computational resources and training time, they often sacrifice the performance of head classes. In contrast, our solution enhances the performance of all classes without complex rebalancing loss.

Evaluation metrics. We primarily use accuracy (ACC) to evaluate our programs. In addition, we report on group assessment measures. ACC is a well-known and widely used evaluation metric for classification tasks. Specifically, the predicted label with the highest probability value is designated as the label for a given sample. ACC-top1 is computed by dividing the number of correctly predicted samples by the total number of samples in the test set. In the absence of any explicit specification, ACC refers to the ACC-top1 across all categories.

Implementation. We train our models using the PyTorch toolbox on GeForce RTX 3090Ti GPUs. The networks are trained for 200 epochs with stochastic gradient descent (SGD) (momentum = 0.9), a weight decay 2 × 10^−4^, and a batch size of 128. The initial learning rate is set to 0.1 with 5 epochs of linear warm-up—the learning rate decays at 120 and 160 by 0.01.

Comparison methods. We compare MMT with a comprehensive baseline approach, including transfer, re-weighting, and augmentation approaches.

- RS (Re-Sampling) [54]: Balancing the objective from different sampling probability for each sample.
- RW (Re-Weighting) [55]: Balancing the objective from different weights on the sample-wise loss.
- CB (Class-Balanced loss) [41]: A novel theoretical framework to measure data overlap by associating a small neighboring region with each sample rather than a single point with each sample.
- Focal loss [44]: It considers the imbalanced distribution of data and the distinguished complex sample. In detail, we replace the cross-entropy loss with focal loss in the experiment.
- LDAM [11]: A theoretically principled label-distribution-aware margin (LDAM) loss motivated by minimizing a margin-based generalization bound.
- BBN (Bilateral-Branch-Net) [14]: A unified Bilateral-Branch Network to take care of both representation learning and classifier learning simultaneously, where each branch performs its duty separately.
- BS (Balanced meta-Softmax) [56]: An elegant unbiased extension of Softmax to accommodate the label distribution shift between training and testing.
- Meta-weight-net [45]: A method capable of adaptively learning an explicit weighting function directly from data.
- IB (Influence-Balanced loss) [42]: A balancing training method to address problems in imbalanced data learning.
- Mixup [20]: Mixup is a classical data augmentation algorithm combining input data and corresponding labels.
- SMOTE (Synthetic Minority Oversampling Technique) [15]: Oversampling minority samples by interpolating between existing minority samples and their nearest minority neighbors.
- Remix (ECCV 2020) [21]: Remix assigns the label in favor of the minority class by providing a disproportionately higher weight to the minority class.
- M2m (Major-to-minor) [13]: A novel yet simple way to alleviate this issue is by augmenting less-frequent categories via translating samples (e.g., images) from more-frequent categories.
- CMO (Context-rich Minority Oversampling) [22]: A method pastes an image from a minority class onto rich-context images from a majority class, using them as background images.
- CUDA (CUrriculum of Data Augmentation) [57]: A method proper degree of augmentation be allocated for each class to mitigate class imbalance problems.

### 4.2. Main Results

In this section, we assess the effectiveness of our proposed method through a series of empirical studies, including the Experimental of CIFAR-100-LT, CIFAR-10-LT, Tiny ImageNet-LT, and Food101-LT.

#### 4.2.1. Results on CIFAR-100-LT

To evaluate the effectiveness of MMT, we initially conducted experiments by training ResNet-32 and ResNet-20 networks on CIFAR-100-LT, considering imbalance ratios of 100, 50, and 10. The results are summarized in Table 2. Across all three imbalance ratios in the long-tailed CIFAR-100-LT datasets, MMT consistently improves overall performance in long-tailed classification. Specifically, MMT achieves classification accuracies of 44.77%, 49.24%, and 61.34% for imbalance ratios of 100, 50, and 10, respectively. Compared to the baseline CE, MMT exhibits performance enhancements of 6.12%, 4.49%, and 4.61% on datasets with varying imbalance ratios. Furthermore, we compare MMT with other transfer-based long-tailed classification methods, i.e., SMOTE, M2m, and CMO. The results indicate that MMT consistently outperforms transfer-based methods. Specifically, MMT outperforms the method M2m by 1.87% and 3.14% for imbalance ratios of 100 and 10, respectively. Furthermore, MMT surpasses the method CMO by 3.2% when the imbalance ratio is 100. The limitations of the above methods lie in restricting the diversity of generated minority samples, as they solely transfer information to the tail classes. The key idea of MMT is to mutually transfer the knowledge between head and tail samples. In pursuit of this objective, MMT not only learns more generalizable features of minority classes by transferring and leveraging the diversity inherent in majority information but also ensures the preservation of majority class features. These improvements underscore the superior performance of MMT, particularly when faced with a high imbalance ratio of 100.

Similarly, as demonstrated in Table 3, MMT consistently outperforms the baseline CE method when employing the ResNet-20 network. Specifically, MMT exhibits performance improvements of 3.97%, 5.34%, and 4.8% across the evaluated imbalance ratios. Moreover, we compare MMT with other long-tailed classification methods, consistently outperforming them significantly. Consequently, regardless of the ratio of data imbalance, MMT consistently improves the model performance across these datasets.

#### 4.2.2. Results on CIFAR-10-LT

We conduct experiments on CIFAR-10-LT using different imbalance ratios: 100, 50, and 10, to demonstrate the effectiveness of our proposed method. The classification accuracy for network ResNet-32 of our method and other methods are summarized in Table 4. It can be observed that MMT boosts the model performance in all different imbalance ratio long-tailed datasets. Specifically, MMT attains accuracy rates of 79.59%, 83.30%, and 90.21% on ResNet-32. As demonstrated in Table 4, MMT consistently outperforms other rebalancing schemes (RW, Balanced meta-Softmax, IB, etc.) across all three imbalance ratios. Concretely, MMT showcases performance improvements of 1.29% and 2.31% compared to M2m. Compared to CMO, MMT achieves higher classification results across the three imbalance ratios with improvements of 5.59%, 2.79%, and 1.34%, respectively.

The performance of MMT is also evaluated on network ResNet-20, and the results are shown in Table 5. Compared to CMO, MMT achieves a significant performance improvement, particularly in the 100 ratios, with a notable improvement of 3.54%. Compared to more complex design schemes, such as the two-branch BBN [14] and the Balanced meta-Softmax [56] based on meta-learning, MMT shows slightly lower performance at the imbalance ratio of 100. In our experiments, we implemented the hybrid MMT + CutMix, which achieved the best performance over the 3 imbalance ratios of 100, 50 and 10, as highlighted in bold in Table 5. Overall, MMT exhibits more stable performance across different imbalance ratios.

#### 4.2.3. Results on Tiny ImageNet-LT

We further evaluate the effectiveness of MMT on the Tiny ImageNet-LT dataset, and the results are presented in Table 6. Accuracy details are provided for both the ResNet-32 and ResNet-20 networks. Comparing MMT to CB and BBN, we observe superior performances achieved by MMT. Specifically, when compared to CB and BBN, we find that BBN outperformed CB by 1% on the ResNet-32 model when the imbalance ratio is 50. Additionally, BBN achieves improvements of 1.61% and 1.29% over CB on the ResNet-20 model for imbalance ratios of 100 and 10, respectively. While the results of the two schemes are very close in other cases, the overall performance of BBN is superior to CB. Notably, MMT consistently outperforms BBN across different models and imbalance ratios, particularly when the imbalance ratio is 50. Specifically, MMT demonstrates improvements of 3.8% and 4.19% over BBN on the networks ResNet-32 and ResNet-20, respectively. Additionally, the results in Table 6 clearly illustrate that CMO exhibits varying degrees of performance degradation compared to the baseline method CE. We speculate that this phenomenon arises from the performance instability caused by the CMO’s lack of consideration for head information and reliance on partial data for oversampling. In contrast, MMT consistently demonstrates superior performance and stability, mainly when dealing with larger imbalance ratios.

#### 4.2.4. Results on Food101-LT

The results of Food101-LT are summarized in Table 7. While the unified Bilateral-Branch Network (BBN) [14] demonstrates enhanced accuracy by concurrently addressing representation learning and classifier refinement, our approach surpasses BBN by 4.33% without necessitating decoupled training, particularly evident when the imbalance ratio is set to 100. In addition, compared with the transfer knowledge from head to tail method CMO [22], the performance is still superior as food image classification is more challenging. The results show the effectiveness of our mutual transfer knowledge between head and tail classes to maintain the learned information and the use of visually aware augmentation for better generalization in all classes.

### 4.3. Further Analysis

Delving into the impact of our method on long-tailed classification, we trained the ResNet-32 network on CIFAR-100-LT (IF = 100), with the results summarized in Table 8. MMT significantly improves tail class accuracy from 8.60% (CE) to 12.67%, while also achieving 71.57% on head classes, surpassing Mixup (71.00%) and CMO (69.54%). For the medium class, MMT reaches 45.49%, leading other methods by a considerable margin. These results demonstrate MMT’s superior ability to handle long-tailed distributions, particularly in boosting Tail class performance.

Based on the results in Table 9, while our method (MMT) requires slightly more training time compared to CUDA (0.14 vs. 0.09 min/epoch) and CMO (0.14 vs. 0.08 min/epoch), it significantly outperforms both in terms of accuracy. Specifically, MMT achieves an accuracy of 44.77%, which is 4.22% higher than CUDA (40.55%) and 3.2% higher than CMO (41.57%). The testing times for MMT are comparable to those of other methods, and memory consumption remains consistent across different approaches. Despite the longer training time, as indicated in Table 8, MMT shows performance improvements across all class groups—many, medium, and few. Compared to CMO and CUDA, MMT delivers the best performance in every category.

Delving a deeper understanding of the impact of our proposed method on long-tailed classification, we present the Top-1 accuracy results of each category in the CIFAR-10-LT (IF = 50) dataset using the network ResNet-32, as shown in Table 10. In particular, the focal loss and CB methods decrease the accuracy of the head classes, i.e., airplane, car, bird, and cat. A similar observation is seen for the LDAM method, i.e., bird and cat. It suggests that the performance improvement achieved by these methods often comes at the expense of compromising the majority of categories. In stark contrast, our proposed approach effectively mitigates the performance degradation within the head category and improves the tail accuracy.

### 4.4. Ablation

#### 4.4.1. Effect of the Dataset Update and Data Sampler in MMT

To demonstrate the effectiveness of the dataset update and data sampler in our proposed method, we compare MMT with Mixup and variant hlMMT *w*/*o* differential sampler. In the Mixup experiment, it is directly employed to augment the long-tailed dataset. The variant MMT *w*/*o* differential sampler samples two samples using a uniform sampler. We conducted experiments by training the ResNet-32 network on the CIFAR-10/100-LT datasets. As illustrated in Table 11, the results consistently indicate that MMT and its variant, MMT *w*/*o* differential sampler, outperform Mixup in long-tailed classification across varying imbalance ratios on CIFAR-10-LT/CIFAR-100-LT dataset. Specifically, on the CIFAR-100-LT dataset, MMT outperforms Mixup by 5.23%, 4.25%, and 3.32% for imbalance ratios of 100, 50, and 10, respectively. Similarly, the variant MMT *w*/*o* differential sampler surpasses Mixup by 3.81%, 4.19%, and 3.27%. The significant improvement demonstrates the importance of data updating in enhancing the performance of long-tailed deep networks. It is noteworthy that MMT outperforms the variant MMT *w*/*o* differential sampler, highlighting the positive impact of the data sampler on model performance. Moreover, MMT significantly improves handling larger imbalance ratios more than smaller ones.

#### 4.4.2. Effect of Fusion Strategy in MMT

One might question whether our proposed method intensifies dataset imbalance and decreases the performance of long-tailed deep networks. To address this concern, we evaluate two variants of MMT, which exclusively generate tail samples. The first variant, MMT_tail_, generates tail samples by making the mixed ratio λ greater than 0.5 in Equation (3). The second variant, MMT_tail-beta_, generates tail samples by controlling Beta distribution, i.e., Beta(1,α). We conduct these experiments using the CIFAR-100-LT dataset and ResNet-32, and the results are presented in Table 12. It is evident that MMT_tail_ and MMT_tail-beta_ lead to an acceptable performance improvement by increasing the sample size of tail samples. However, this improvement is far less than that achieved by the original MMT. Both variants, MMT_tail_ and MMT_tail-beta_, only transfer knowledge from head to tail. Therefore, the head samples lack the regularization effect of tail information, resulting in a limited performance boost. The disparity in the accuracy of head classes between the original MMT and its variants underscores this point.

#### 4.4.3. Effect of Hyperparameter α of Beta Distribution

To better understand the effect of the hyperparameter α (Equation (3)) in our proposed method, we conduct ablation on the hyperparameter α. The hyperparameter α is introduced to control the ratio of the two mixed samples. We adopt ResNet-32 to explore a range of α values, including 0.1, 0.2, 0.5, 0.7, and 1.0, on the CIFAR-10-LT dataset with three different imbalance ratios (IF = 100/50/10). The evaluation results are presented in Table 13. We observe that 0.5 yields better performance than other values on imbalance ratios 50 and 10. The value 0.2 yields the best results on an imbalance ratio of 100. Considering the degree of imbalance ratio, we adopt an α value of 0.2 for all our experiments.

## 5. Conclusions

In this study, we propose a novel method, mixed mutual transfer (MMT), to improve long-tailed classification by facilitating bidirectional knowledge transfer between head and tail classes. By employing a unique sample blending strategy that combines samples from a uniform sampler and a differential sampler, MMT creates diverse and representative training samples, effectively improving the performance of deep neural networks on both head and tail classes. Extensive experiment results on various imbalanced datasets demonstrate that our proposed method exhibits superior classification performance compared to other methods in handling long-tailed data. Additionally, the ablation studies illustrate the effectiveness of our proposed method. In the future, we will explore a more practical approach to simplify the mixed mutual transfer procedure where the generated samples increase the training cost.

## Figures and Tables

**Table 1:** Summary of datasets.

Dataset	Training Samples	Categories	Image Size	Testing Samples	Imbalance Ratio (IF)
CIFAR-10	60,000	10	32×32	10,000	{10, 50, 100}
CIFAR-100	60,000	100	32×32	10,000	{10, 50, 100}
Tiny ImageNet	100,000	200	64×64	10,000	{10, 50, 100}
Food 101	101,000	101	64×64	10,000	{10, 50, 100}

**Table 2:** Comparison on CIFAR-100-LT using ResNet-32. Top-1 test accuracy (%) for ResNet-32 network on CIFAR-100-LT with different imbalance ratios. † indicates that results are obtained from experiments in M2m [13]. The notation ‡ represents results directly sourced from [42], while ‘*’ signifies results replicated from [45]. Solutions not marked by these symbols are derived from our own reproduced results. The best results are highlighted in bold.

	IF = 100	IF = 50	IF = 10
Baseline (CE)	38.65	44.75	56.73
RS [54] ^†^	31.60	-	54.80
RW [55] ^†^	31.10	-	56.00
Focal loss [44] ^‡^	38.41	44.32	55.78
SMOTE [15] ^†^	34.00	-	49.40
CB (CVPR 2019) [41]	38.45	43.27	56.82
DRW (NeurlPS 2019) [11]	41.02	46.74	57.65
Balanced meta-Softmax (NeurlPS 2020) [56]	41.87	46.59	57.79
LDAM + DRW (NeurlPS 2019) [11]	42.43	46.92	57.22
IB (NeurlPS 2021) [42] ^‡^	42.14	46.22	57.13
Meta-weight Net(2019) [45] *	42.09	46.74	58.46
BBN (CVPR 2020) [14]	40.62	44.32	58.61
Remix (ECCV 2020) [21]	41.94	-	59.36
M2m (CVPR 2020) [13] ^†^	42.90	-	58.20
CMO (CVPR 2022) [22]	41.57	47.83	59.54
CUDA (ICLR 2023) [57]	40.55	45.12	58.35
MMT	**44.77**	**49.24**	**61.34**

**Table 3:** Comparison on CIFAR-100-LT using ResNet-20. Top-1 test accuracy (%) for ResNet-20 network on CIFAR-100-LT (IF = 100/50/10).

	IF = 100	IF = 50	IF = 10
Baseline (CE)	39.07	42.59	55.38
CB [41]	39.05	42.61	55.43
DRW [11]	41.16	46.09	57.97
LDAM + DRW [11]	41.85	46.74	55.64
Balanced meta-Softmax [56]	42.72	46.91	56.87
BBN [14]	40.68	44.72	56.32
CMO [22]	40.91	46.75	58.40
MMT	43.04	47.93	60.18

**Table 4:** Comparison on CIFAR-10-LT using ResNet-32. Top-1 test accuracy (%) evaluated on CIFAR-10-LT (IF = 100/50/10) with ResNet-32 backbone. † indicates that results are obtained from experiments in M2m [13]. The notation ‡ represents results directly sourced from [42], while ‘*’ signifies results replicated from [45]. Solutions not marked by these symbols are derived from our own reproduced results. The best results are highlighted in bold.

	IF = 100	IF =50	IF = 10
Baseline (CE)	70.86	76.53	86.59
RS [54] ^†^	70.40	-	85.70
RW [55] ^†^	72.80	-	86.60
Focal loss [44] ^‡^	70.38	76.71	86.66
SMOTE [15] ^†^	71.50	-	85.70
CB [41]	72.08	76.59	86.76
DRW [11]	75.58	80.30	87.53
LDAM + DRW [11]	77.70	81.57	87.78
Balanced meta-Softmax [56]	77.10	81.20	88.23
IB [42] ^‡^	78.26	81.70	88.25
Meta-weight Net [45] *	75.21	80.06	87.84
BBN [14]	78.26	81.20	87.54
Remix [21]	75.36	-	88.15
M2m [13] ^†^	78.30	-	87.90
CMO [22]	74.00	80.51	88.87
CUDA [57]	74.83	77.87	86.57
MMT	**79.59**	**83.30**	**90.21**

**Table 5:** Top-1 test accuracy (%) evaluated on CIFAR-10-LT (IF = 100/50/10) with ResNet-20 backbone.

	IF = 100	IF = 50	IF = 10
Baseline (CE)	70.71	76.27	86.36
Class-Balanced loss [41]	71.17	75.83	85.61
DRW [11]	76.65	79.94	87.53
LDAM + DRW [11]	76.91	81.69	87.78
BBN [14]	77.43	81.08	87.52
Balanced meta-Softmax [56]	77.17	81.16	87.56
CMO [22]	73.41	79.01	86.69
MMT	76.95	82.13	89.18
MMT + Cutmix	78.95	83.29	89.90

**Table 6:** Comparison on Tiny ImageNet-LT. Top-1 test accuracy (%) evaluated on Tiny ImageNet-LT with ResNet-32 and ResNet-20.

	ResNet-32			ResNet-20		
	IF = 100	IF = 50	IF = 10	IF = 100	IF = 50	IF = 10
Baseline (CE)	25.31	27.80	35.95	23.40	26.66	33.27
CB [41]	25.89	28.11	35.95	23.82	26.70	34.05
BBN [14]	25.83	29.17	36.81	25.43	27.35	35.34
LDAM + DRW [11]	28.31	30.59	35.35	26.98	30.18	33.76
CMO [22]	24.32	27.07	34.13	22.62	24.47	31.89
MMT	29.58	32.97	39.30	28.55	31.54	37.25

**Table 7:** Comparison on Food101-LT. Top-1 test accuracy (%) evaluated on Food 101-LT with ResNet-50.

	IF = 100	IF = 50	IF = 10
Baseline(CE)	45.36	51.54	71.66
BBN [14]	47.76	54.29	73.50
CMO [22]	48.08	57.26	73.97
MMT	52.09	59.39	74.90

**Table 8:** Top-1 test accuracy (%) CIFAR-100-LT (IF = 100).

	All	Head	Med	Tail
Baseline (CE)	38.44	65.09	37.37	8.60
Mixup [20]	39.54	71.00	40.90	4.90
CMO [22]	41.92	69.54	40.03	11.90
CUDA [57]	40.55	69.00	39.23	8.90
MMT	44.77	**71.57**	45.49	12.67

**Table 9:** Training and testing times (CIFAR-100-LT (IF = 100)).

Methods	Training (m/epoch)	Testing (s/epoch)	Acc (%)
Baseline (CE)	0.06	0.65	38.65
Mixup [20]	0.07	0.67	39.54
CMO [22]	0.08	0.67	41.57
CUDA [57]	0.09	0.68	40.55
MMT	0.14	0.66	44.77

**Table 10:** Class-wise classification accuracy on CIFAR-10-LT (IF = 50) with ResNet-32 backbone.

Class	Plane	Car	Bird	Cat	Deer	Dog	Frog	Horse	Ship	Truck
Training samples	5000	3237	2096	1357	878	568	368	238	154	100
Baseline (CE)	96.20	97.70	87.20	78.00	80.80	65.20	78.80	64.90	59.20	57.10
Focal Loss [44]	91.60	95.10	73.10	59.20	67.80	67.20	84.20	77.30	83.90	61.80
CB [41]	92.90	96.30	79.20	75.10	82.40	69.90	75.00	69.10	73.60	66.80
LDAM [11]	96.90	98.50	82.90	74.70	82.80	69.00	78.50	69.90	65.30	66.00
MMT	96.40	98.90	91.70	82.40	86.40	74.60	86.10	77.50	73.20	65.50

**Table 11:** Ablation studies on the effectiveness of data sampler on CIFAR-100/10-LT. The network ResNet-32 is used.

	CIFAR-100-LT			CIFAR-10-LT		
	IF = 100	IF = 50	IF = 10	IF = 100	IF = 50	IF = 10
Mixup [20]	39.54	44.99	58.02	73.06	77.82	87.10
MMT *w*/*o* differential sampler	43.35	49.18	61.29	77.10	81.44	89.88
MMT	44.77	49.24	61.34	79.59	83.30	90.21

**Table 12:** Ablation studies of different fusion strategies on the CIFAR-100-LT (IF = 100) dataset (ResNet-32).

	All	Head	Medium	Tail
Baseline (CE)	70.86	65.09	37.37	8.60
MMT_tail_	77.45	69.31	45.63	12.57
MMT_tail-beta_	77.80	69.74	44.40	12.80
MMT	79.59	71.57	45.49	12.67

**Table 13:** Ablation studies on hyperparameter α of Beta distribution. The CIFAR-10-LT and ResNet-32 are adopted.

α	IF = 100	IF = 50	IF = 10
0.1	78.15	82.06	90.08
0.2	79.59	83.30	90.21
0.5	78.57	83.34	90.30
0.7	78.17	82.56	89.99
1.0	77.81	83.24	89.93

## Data Availability

Data are available in a publicly accessible repository.
